# Correction: Eyewitness accuracy and retrieval effort: Effects of time and repetition

**DOI:** 10.1371/journal.pone.0307692

**Published:** 2024-07-18

**Authors:** Philip U. Gustafsson, Torun Lindholm, Fredrik U. Jönsson

There are errors in the Author Contributions. The correct contributions are:

**Conceptualization:** Philip U. Gustafsson.

**Formal analysis:** Philip U. Gustafsson.

**Funding acquisition:** Philip U. Gustafsson, Torun Lindholm.

**Investigation:** Philip U. Gustafsson.

**Methodology:** Philip U. Gustafsson, Torun Lindholm, Fredrik U. Jönsson.

**Project administration:** Philip U. Gustafsson.

**Resources:** Philip U. Gustafsson, Torun Lindholm.

**Supervision:** Torun Lindholm, Fredrik U. Jönsson.

**Visualization:** Philip U. Gustafsson.

**Writing–original draft:** Philip U. Gustafsson.

**Writing–review & editing:** Philip U. Gustafsson, Torun Lindholm, Fredrik U. Jönsson.
