# Supplementary material for: Eyewitness accuracy and retrieval effort: Effects of time and repetition
Source: PLoS One. 2022 Sep 7;17(9):e0273455. doi: 10.1371/journal.pone.0273455 (PMC9451081; doi:10.1371/journal.pone.0273455)

Supplementary material
Gustafsson et al.
Eyewitness Accuracy and Retrieval Effort: Effects of Time and Repetition
PLOS ONE

Fig S2. Effects of accuracy, time and repetition on confidence and the retrieval-effort index in free and cued recall. Error bars represent 95 % confidence intervals.

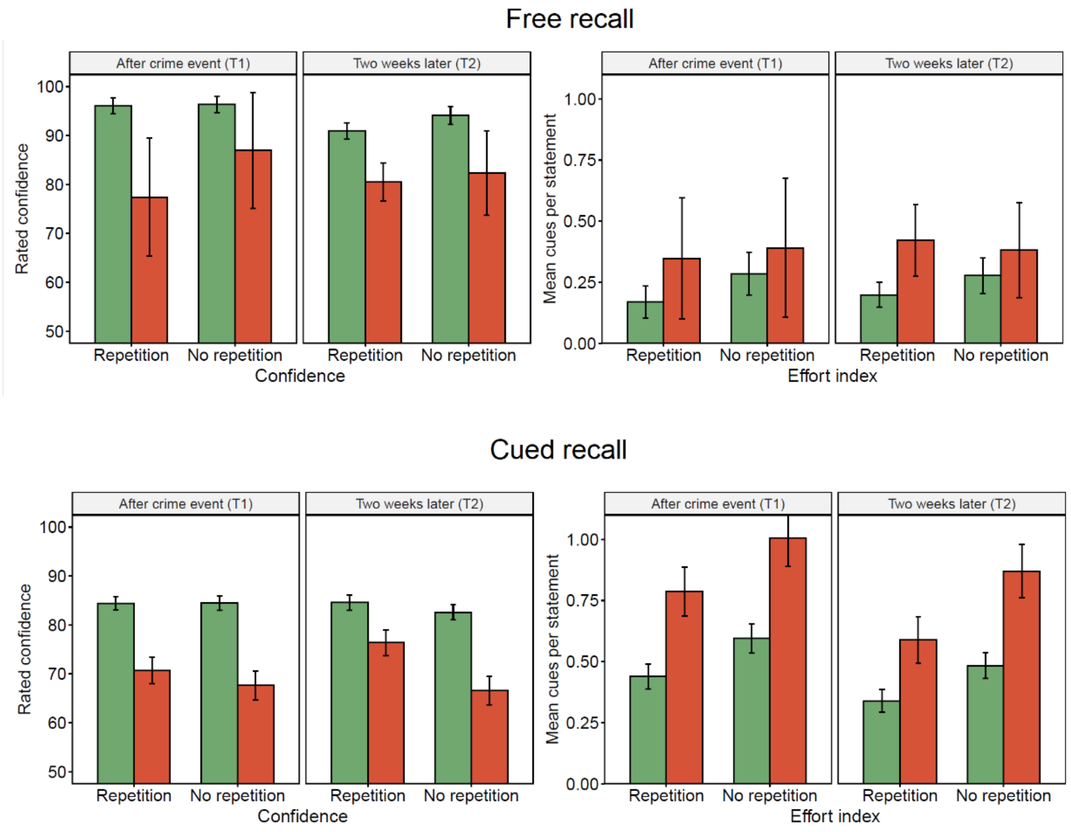

Supplement: S2 Fig — (PDF) [file pone.0273455.s002.pdf]
