# Supplementary material for: Eyewitness accuracy and retrieval effort: Effects of time and repetition
Source: PLoS One. 2022 Sep 7;17(9):e0273455. doi: 10.1371/journal.pone.0273455 (PMC9451081; doi:10.1371/journal.pone.0273455)
Supplement: S1 File — (PDF) [file pone.0273455.s003.pdf]

File S1. Analyses of total amount of statements in testimonies.

An ANOVA with *Total statements* showed no significant effect of *Time*, $F(1, 54) = 2.29$, $p = .133$, $\eta^2 = .019$ (see Table 3). However, the effect of *Repetition* was significant, such that the repetition group provided a greater amount of statements ($M = 77.87$, $SD = 18.17$) compared to the no-repetition group ($M = 70.40$, $SD = 19.15$); $F(1, 54) = 4.64$, $p = .034$, $\eta^2 = .039$ (see Table 3). The interaction between *Time* and *Repetition* was not statistically significant, $F(1, 54) = 3.72$, $p = .056$, $\eta^2 = .031$. As with *Unique details* in the main manuscript, we carried out planned comparisons between the repetition group and the no-repetition group at T2. Results showed a significantly greater amount of *Total statements* for the repetition group; $M_{diff} = 14.17$, $p = .034$, $d = 0.68$ (see Table 3).

File S1 Table. Means (and standard deviations) for total amount of statements across accuracy, time and repetition.

		Total statements	
		Repetition group	No-repetition group
After crime event (T1)	Correct	55.37 (11.09)	54.21 (15.33)
	Incorrect	16.41 (5.46)	16.79 (6.53)
	Total	71.78 (12.64)	71.00 (19.20)
Two weeks later (T2)	Correct	61.15 (15.48)	52.41 (15.56)
	Incorrect	22.81 (10.71)	17.38 (7.54)
	Total	83.96 (20.89)	69.79 (19.42)
