# Supplementary material for: Eyewitness accuracy and retrieval effort: Effects of time and repetition
Source: PLoS One. 2022 Sep 7;17(9):e0273455. doi: 10.1371/journal.pone.0273455 (PMC9451081; doi:10.1371/journal.pone.0273455)
Supplement: S1 Table — (PDF) [file pone.0273455.s004.pdf]

Supplementary material
Gustafsson et al.
Eyewitness Accuracy and Retrieval Effort: Effects of Time and Repetition
PLOS ONE

Table S1. Percentage distribution of ratings for each level of confidence across accuracy, time, and repetition.

	After crime event				Two weeks later			
	Repetition		No repetition		Repetition		No repetition	
Confidence level	Correct	Incorrect	Correct	Incorrect	Correct	Incorrect	Correct	Incorrect
100	60.71 %	25.59 %	63.58 %	30.54 %	62.39 %	28.32 %	58.66 %	25.85 %
80	19.09 %	30.59 %	18.79 %	23.51 %	18.13 %	28.50 %	21.27 %	30.29 %
60	13.31 %	24.71 %	0.89 %	21.08 %	12.93 %	21.03 %	11.23 %	17.23 %
40	4.84 %	13.24 %	4.99 %	12.43 %	4.67 %	7.71 %	5.25 %	14.62 %
20	1.68 %	4.41 %	3.47 %	9.46 %	1.89 %	3.74 %	2.76 %	9.92 %
0	0.19 %	1.47 %	0.27 %	2.97 %	0 %	0.07 %	0.83 %	2.09 %
