# Supplementary material for: Eyewitness accuracy and retrieval effort: Effects of time and repetition
Source: PLoS One. 2022 Sep 7;17(9):e0273455. doi: 10.1371/journal.pone.0273455 (PMC9451081; doi:10.1371/journal.pone.0273455)
Supplement: S2 Table — (PDF) [file pone.0273455.s005.pdf]

Supplementary material
Gustafsson et al.
Eyewitness Accuracy and Retrieval Effort: Effects of Time and Repetition
PLOS ONE

Table S2. Mediations between confidence and accuracy for each effort cue.

	Percentage mediated
Delays	5.41 %
Hedges	23.00 %
Non-word fillers	< 0.01 %
Word fillers	< 0.01 %
False starts	< 0.01 %
Prolongations	< 0.01 %
