# Supplementary material for: Eyewitness accuracy and retrieval effort: Effects of time and repetition
Source: PLoS One. 2022 Sep 7;17(9):e0273455. doi: 10.1371/journal.pone.0273455 (PMC9451081; doi:10.1371/journal.pone.0273455)
Supplement: Attachment — Submitted filename: Response to reviewers.docx [file pone.0273455.s006.docx]

Responses to reviewer comments.

Thank you for your review of our submission, “Eyewitness Accuracy and Retrieval Effort: Effects of Time and Repetition” to PLOS One. As in the following, we have addressed the helpful comments and revised the paper. We hope that all the corrections and revisions will be satisfactory.

Please note that new text is marked with italics, and numbered lines refer to lines in the manuscript with tracked changes turned off.

**Reviewer 1**

1. Coverage of the relevant literature.

There are some relevant, and important, aspects of the literature that are omitted from the introduction. When considering factors associated with recall memory performance, it is really important to consider how metacognitive monitoring and control processes support the retrieval and reporting of information. We know that participant witnesses will strategically regulate report option (i.e., the decision to report or withhold retrieved information) and grain size/precision (i.e., the amount of detail reported) to balance competing demands for informativeness (provide as much information as possible) and accuracy (make the information you provide as reliable as possible). These metacognitive control processes affect the amount and accuracy of information volunteered (e.g., Fisher, 1996; McCallum et al., 2016), and confidence in the accuracy of volunteered information (with different confidence-accuracy relationships evident for fine- and coarse-grained details; e.g., Sauer & Hope, 2016; Weber & Brewer, 2008). Even if the researchers don’t want to include this as a design or analytical element of the current work, some consideration of how things like retrieval effort relate to (and may feed into) these underlying monitoring and control processes seems relevant.

**Response:** We agree that this is informative to the reader and have added the following section in the introduction, lines 186-194:

*It is also important to highlight that the increased effort to report memories that have been weakened over time may be offset by changes in grain-size reporting (see e.g. Goldsmith et al., 2002; McCallum et al., 2016; Weber & Brewer, 2008). For example, if the memory of a fine-grained detail (e.g. “green sport jacket”) becomes weakened over time (leading to a more effortful retrieval), the witness may opt to report a more coarse-grained version of that detail instead (e.g. “jacket”). The expected increased effort to report the fine-grained details of that memory is therefore likely lost, resulting in a more easily retrieved memory. It is therefore possible that memory quality may be diminished over time due to a loss of detail, whereas the quantity might remain constant (see Fisher, 1996), as well as the retrieval effort.*

2. Based on this literature, there are three implications for the current work (and, I think, for any work wishing to thoroughly understand the effects of manipulations on, and reliable indices of, recall accuracy):

First, ideally, the authors would code responses to provide details on how their manipulations affected the proportion of fine- and coarse-grained details provided. Second, ideally, markers of recall accuracy would be assessed differently for fine- and coarse-grained details. Finally, at a minimum, it is important to know (a) what instructions participants were given relating to the regulation of report option and grain size [were participants instructed, implicitly or explicitly, to prioritise accuracy or informativeness], and (b) how were answers coded for accuracy? For example, if the target wore a black top, would a response saying the top was “dark” be coded as correct or incorrect? Either approach is defensible – and there is certainly variability on how this issue has been treated in the past – so it’s important that reader (and those who may wish to replicate the work later) are clear on these details.

**Response:** We agree that the question about grain size and effort is interesting and should be examined. At the same time, we believe that such an examination deserves its own focus, instead of added here as an additional analysis. We have added a line in the discussion that highlights such an examination, and encourage other researches to carry out such coding and analysis of our data, lines 599-606:

*The third explanation is a shift in grain-size of the details. Koriat and Goldsmith (1996) suggests that people can not only decide to withhold or report a memory, but also shift the level of detail with which the memory is reported. Thus, a piece of clothing could both be described with a high level of detail (“a blue zip up jacket with green stripes and a hood”) or a low level of detail (“a jacket”). In this study we did not code for grain-size, so it is possible that the repetition group remembered things in more detail than did the no-repetition group. We encourage researchers to examine this in future studies, and as our data is openly available, suggest examinations also of our data.*

Regarding (a), we have clarified the exact instructions given to the participants, which involved no overt instruction about grain size reporting, lines 253-256:

The interview started with an open free recall task *(“I would like you to start by freely recounting what you have seen”*) followed by open direct questions (e.g. “How was the first perpetrator dressed?”). *No specific instruction regarding grain size of reporting was given.*

Regarding (b), we have clarified the coding of accuracy, as well as the coding of statements, lines 283-297:

For example, the statement “he had a green coat” is an objectively verifiable detail, whereas “he was handsome” is not. *Responses that contained several details pertaining to the same object/event were coded as one statement when produced in a clustered fashion (e.g. “He wore dark blue jeans”), and coded as independent statements when produced in a dispersed fashion (e.g. “He wore jeans.” // “I think they were dark blue”).* The coders first coded 10 % of the material together (exact overlap = 82.19 %), and one coder then coded the remaining 90 %. Next, the same two coders coded memory accuracy. *Statements were only coded as correct if they contained wordings that correctly matched the description in the coding sheet. For certain details, several accepted terms were provided in the coding sheet (e.g. hair color as “black” and “dark”, pants color as “dark”, “dark blue”, and “black”). Adverbs of degree (e.g. “quite”, “somewhat”) were to be ignored in the judgment about accuracy, unless specifically stated in the coding sheet. Statements that did not match the wording in the coding sheet (e.g. “light color” for a detail that was “white”) were excluded.* Statements that contained both correct and incorrect details were also excluded(e.g. *“He had a black beard”*, where “black” = correct, and “beard” = incorrect).

3. Further, in general, when motivating the research and considering the implications of findings, the authors need to be clearer in distinguishing between confidence and accuracy for recognition memory tasks and confidence and accuracy for recall memory tasks. There are similarities in the theoretical mechanisms at play, but there are also important differences. For example, inferential cues are prominent in theoretical explanations for confidence in recall (and associated metacognitive domains), but theories used to describe confidence processing for recognition tasks draw more heavily on signal detection theory and evidence accumulation models; emphasising the strength of the evidence underlying the decision rather than participants’ perceptions of the decision process.

**Response:** We now mainly present evidence for recall tasks in the introduction. If recognition evidence is provided, this is overtly stated, and a comment or supporting recall study is provided.

Lines 58-64:

There are now many studies that attest to a relationship between retrieval effort and accuracy, in which correct memories tend to be retrieved more fluently than incorrect memories. For example, when participants in two experiments by Brewer, et al. (2006) watched a staged crime and had to identify the culprit in a line-up, those who made correct identifications responded quicker than those who made incorrect identifications. Similarly, *in a recall study*, Smith and Clark (1993) showed that correct verbal responses to general knowledge questions were quicker […]

Lines 121-132:

*With time, people risk becoming overconfident, resulting in a diminished confidence-accuracy relationship. For example,* an initial confidence judgment (e.g. “I’m not very confident”) may accurately reflect identification performance (i.e. identified the wrong person), but through the course of time, *a witness may become certain (“I’m very confident”), but the accuracy is not likely to change (i.e. still identifies the wrong person). This is not just a hypothetical example, as evidenced from several real-life cases of mistaken eyewitness identifications (see Garrett, 2011), in addition to lab experiments (Palmer et al., 2013; Sauer et al., 2010; Wixted et al., 2016). The increased confidence over time has also been shown in recall studies (Chandler, 1994; Odinot, et al., 2009; Shaw, 1996; Shaw & McClure, 1996, cf. Odinot, et al., 2013; Knutsson, et al., 2011), thus showing a similar pattern for both memory recall and memory recognition. Indeed, the confidence-accuracy relationship appear similar across both recall and recognition (Robinson et al., 1996).*

Lines 179-183:

*For example, leading questions (e.g. Loftus & Palmer, 1974) and post-event feedback (Gurney et al., 2014; for identification research, see meta-analysis by Douglass & Steblay, 2006) may bolster one’s belief that a memory is correct, and increase the chances that it is reported (and subsequent confidence judgment).*

4. I really like the use of the multi-level modelling approach, and applaud the authors on using it. However, I think it will be unfamiliar to many readers of the eyewitness recall literature where repeated measures ANOVAs are still the “norm”. Thus, when presenting the results in Table 3, it may help readers appreciate the meaning of these findings if the authors provide some concrete guidance on interpreting the coefficients in the table. For example, for someone not familiar with interpreting logistic regression (or multi-level modelling), what do the data tell us about how the likely accuracy of a detail varies according to changes in the “hedges” variable (other than the obvious interpretation that the relationship is negative).

**Response:** Thank you for this suggestion. To facilitate comprehension, we have added a column with “unstandardized odds ratios”, which are easier to interpret than standardized odds ratios (see Table 2). Standardized values are kept as they facilitate comparisons between variables on different scales (such as hedges vs. confidence). We have also added a descriptive text to facilitate reading comprehension, lines 427-433:

In Table 2, the odds ratio indicates the increase/decrease in accuracy when increasing one step on the scale of each variable, with values above zero indicating an increase, and values below zero indicating a decrease. That is, a statement with no hedges will be 29% more likely to be correct compared to a statement with one hedge (UOR = 0.71), and a confidence judgment with 81% confidence is 3 % more likely to be correct compared to a confidence judgment of 80 % (UOR = 1.03; see Table 2).

5. On a similar note, the results section contains a lot of output, for some reasonably complex models. I think it would help readers process this output, and appreciate the implications of the reported results, if the authors yoked the presentation of results to specific hypotheses/research questions. “We hypothesised, X. If this hypothesis were borne out in the data, we would expect to see…” then you can present the outputs and it becomes very easy for the reader to appreciate how the reported outputs align with expectations. I think steps like these become increasingly important when presenting unfamiliar analyses.

**Response:** We have now added more detailed descriptions of the multilevel process (and hypotheses) to facilitate comprehension.

Lines 397-401:

*We expected fewer effort cues and greater confidence for correct memories (Hypothesis 1). If this hypothesis were to be supported in the data, we would expect a) that predictor models would differ significantly from baseline-models, and b) have a greater Akaike weight ( > .5) than the baseline model (for more on Akaike weights, see Burnham & Anderson, 2002, 2004).*

Line 426:

*No explicit prediction was made for this analysis.*

Lines 447-470:

To test the effect of time and repetition on retrieval effort, we compared a baseline, intercept-only model of the effort index with models containing *Time*, *Repetition*, *Accuracy* and their interactions as predictors. *For these analyses we expected a main effect of accuracy (a greater effort index value for incorrect memories, Hypothesis 1), and then several interactions (Hypotheses 2-5). In short, these interactions could be summarized as an easier retrieval over time for the repetition group and a more difficult retrieval over time for the no-repetition group (Hypothesis 2-3), and a smaller difference in retrieval effort between correct and incorrect memories over time for the repetition group (Hypothesis 4-5). As in the previous multilevel analyses, we would expect these differences to manifest in the data as significant predictor models with higher Akaike weights than baselines models. Turning to the results then, analyses showed that that model fit was indeed significantly improved compared to the baseline model when adding Accuracy,* such that incorrect statements (*M* = 0.77, *SD* = 0.97) were produced with more effort compared to correct statements (*M* = 0.42, *SD* = 0.75; *d* = 0.44; see Table 3 and Fig 4). *Unexpectedly,* model fit was also significantly improved when adding *Time,* such that statements were produced with more effort at the first interview (T1; *M* = 0.57, *SD* = 0.88) compared to the second interview two weeks later (T2; *M* = 0.45, *SD* = 0.77, *d* = 0.14; see Table 3 and Fig 4). *Moreover,* model fit was significantly improved with *Repetition*, such that the repetition group reported memories with less effort (*M* = 0.42, *SD* = 0.73) compared to the no-repetition group (*M* = 0.59, *SD* = 0.90, *d* = 0.23, see Table 3 and Fig 4). To test interaction effects, we created models containing each interaction and compared them to models of only their respective predictors (e.g. a model with time and accuracy as predictors was compared to a model with time, accuracy and the time-accuracy interaction as predictors*). Here we expected significant interactions of all four combinations of Accuracy, Time and Repetition. Contrary to expectations however, none of the interactions significantly improved fit (see Table 3).*

Lines 476-496:

We then carried out identical analyses for a confidence model. *Expectations were identical to those for retrieval effort but with reversed directions, such that we expected lower confidence in conditions where we had expected higher retrieval effort. In line with predictions,* results showed that model fit was significantly improved compared to the baseline model when adding *Accuracy*, such that confidence was higher for correct statements (*M* = 86.25, *SD* = 21.00) compared to incorrect statements (*M* = 71.65, *SD* = 26.18, *d* = 0.70, see Table 3 and Fig 3). Moreover, *also as expected*, model fit was not significantly improved when adding *Time*, (*M_T1_* = 82.45, *SD* = 23.54; *M_T2_* = 82.54, *SD* = 23.13 *d* < 0.01, see Table 3 and Fig 3); nor *Repetition*, (*M_repetition_* = 83.53, *SD* = 21.73; *M_no-repetition_* = 81.47, *SD* = 24.78, *d* = 0.09; see Table 3 and Fig 3). Interestingly, all interactions improved fit *as expected*: *Time*-*Repetition*, *Time-Accuracy,* *Repetition-Accuracy,* and also *Time*-*Repetition-Accuracy* (see Table 3 and Fig 3). Planned comparisons (see preregistration) showed that confidence *indeed* significantly increased from T1 to T2 for the repetition group (*M_diff_* = 1.62, *p* = .042, *d* = 0.07) whereas there was a nonsignificant decrease for the no-repetition group (*M_diff_* = -1.56, *p* = .087, *d* = 0.06). Moreover, *as expected*, the increased confidence for the repetition group was mainly driven by higher confidence in incorrect statements (*M_diff_* = 6.51, *p* < .001, *d* = 0.27) as there was no significant increase in correct statements (*M_diff_* = 0.53, *p* = .536, *d* = 0.03). For the no-repetition group, the decrease in confidence between T1 and T2 was not statistically significant for neither incorrect statements (*M_diff_* = -0.61, *p* = .764, *d* = 0.02), nor correct statements (*M_diff_* = -1.58, *p* = .091, *d* = 0.07). Hence, in contrast to the results for the effort index, the results for confidence were more in line with expectations.

6. Also: Was participant included as a random factor in these models? Did the models allow for both random intercepts and random slopes, or only for random intercepts?

**Response:** Yes, participant was included as random factor in the model, lines 391 – 392:

We used multilevel modelling with statements nested within witnesses

We have now added a clarification to this sentence, lines 394-396:

We used multilevel modelling with statements nested within witnesses (*i.e. participant as random factor*; Wright & London, 2009)

We only used random intercepts in our model, as random slopes caused overfitting issues.

7. I’m not sure it’s appropriate to group free and cued recall outputs together for analyses. These are different types of memory task, likely to rely differently on the underlying metacognitive monitoring and control processes, and requiring (and potentially facilitating) different levels of effort by the retriever. Collapsing these data may be fine, but some justification for this analytical choice is required.

**Response:** We agree that free and cued recall might yield different output, and that there is good reason to investigate it. Our choice to combine the two was made in order to limit the complexity of the research design, as it already includes the effects of three different variables (and their interactions) on a total of eight outcome variables (six effort cues, confidence, and the effort index). However, in order to give the reader some information about this, we have opted to do the following:

1. Added separate analyses for free, cued and total recall on number of recalled details, including a figure of these findings (see lines 338-388).

Figure (“Fig 1”) inserted here:

2. Add separate figures for free and cued recall for all effort cues, confidence and the effort index as supporting information (see lines 318-319).

*For the interested reader, posteriori plotting of free and cued recall results separately are found in the supplementary section (S 1-2 Figs).*

Figures (“S1 Fig”, “S2 Fig”) inserted here:

As a short comment, it appears from the free/cued recall split figures of confidence and effort index that the time-accuracy-repetition interaction is largely similar across the two recall tasks. The main effect seems to be higher confidence and lesser retrieval effort for free recall.

For transparency, we have also clarified our intentions about free/cued recall on lines 316-320:

For all analyses *of retrieval effort cues and accuracy,* we *decided a priori to* group together free recall and cued recall responses *(see preregistration). This was done to limit the complexity of the research design, which already consisted of three-way interactions on multiple outcome variables (see Hypotheses 3-5). For the interested reader, posteriori plotting of free and cued recall results separately are found in the supplementary section (S1-2 Figs).*

8. Discussion and interpretation

Although I don’t necessary disagree with the authors’ interpretations of their findings, I was struck by the absence of early, explicit consideration of the failed manipulations. The authors need to be careful not to over-interpret their null effects (e.g., of repetition and retention interval on memory performance) and, instead, think about how the absence of these predicted effects might place important caveats on the other conclusions they draw (e.g., relating to effects on effort, confidence, and accuracy). It is a little odd that neither of these typically robust mechanisms showed any effects on recall performance here. Why might that be / what might that indicate? Again, I’d recommend reading Fisher’s (1996) paper in Behavioural and Brain Sciences. It’s a great example of how a seemingly failed manipulation might indicate something of interest in the data. Further, I was interested by the fact that, in the limitations section, there was no mention of the fact that the two key manipulations failed to produce any appreciable effect on memory performance (i.e., the quantity or accuracy of recalled information). This seems like something worthy of comment. In the introduction, the authors set up the paper as an investigation of how time and repetition may affect the relationship between accuracy and memory retrieval effort based on the weakening (via increased retention interval) or strengthening (via repetition) of memories. Moreover, as the authors note in their conclusion: They set out with a special focus on the effects of time and repetition. Thus, their unsuccessful manipulations of both time and repetition would seem to constrain their ability to address the key foci of the experiment. This is not to say that other aspects of the data can’t provide a valuable contribution to the literature; but I’d expect some consideration of these failed manipulations, and how they might colour interpretations of the observed effects.

**Response:** We agree that we should be more cautious in our conclusions, given the lack of forgetting in the no-repetition group. We now believe that the absence of forgetting in the no-repetition group is largely due to three clear retrieval opportunities, taking place at the first interview session. That is, the participants were asked to retrieve their memories of the event at the first interview at both 1) the free and 2) the cued recall. Moreover, another retrieval opportunity was given when the participants directly after the interview 3) rated their confidence, as the interviewer read aloud the respective detail that the participants had recalled. This allowed the participants to elaborate on each detail. Together with likely effects of context-dependent learning (given that the interview took place in the same lab), these effects probably explain the high level of remembrance at the second session. This could also explain why there wasn’t huge differences between the repetition and no-repetition group.

Thus, a better approach to study forgetting over time would have been to skip the recall at the first session and only test it two weeks later. However, our main interest was the effects of time and repetition on *expression of memory* (i.e. retrieval effort cues), rather than amount of memory recall *per se*. And since there appears to be some between-group differences in the usage of retrieval effort cues (see the baseline differences in this study, and also the results in Gustafsson et al., 2021), the effects of time and repetition are arguably preferably studied with a within-group design, such as we have done.

To conclude, we believe that it would be incorrect to see the manipulation of Time as failed, as our results show what *does* happen with recollection and effort two weeks after having viewed an event, but with the qualification that it is based on the rememberer having an initial retrieval attempt.

Moreover, we would argue that our manipulation of Repetition was successful, for several reasons. Firstly, participants in the repetition groups rated themselves as having thought more often (*d* = 1.56) and spent more time thinking about the event (*d* = 0.82). Secondly, although the difference in amount of unique details between the repetition and no-repetition group was not statistically significant (*p* = .052), the effect size was still notable (*d* = 0.52), classified as medium-sized according to Sawilowsky’s (2009) revision of Cohen’s rules of thumb. We place greater emphasis on effects rather than significance (e.g. Rosnow & Rosenthal, 2009; Sullivan & Feinn, 2012; Wilkinson and APA task force, 1999) and therefore deem it fair to consider the manipulation successful. Thirdly, as evident from the new separate free/cued recall analyses (lines 338-388), the repetition group also reported more details in the free recall session two weeks later compared to the no-repetition group (see Figure above). With that said, we do agree that the effects were not as big as we had expected (as mentioned, likely attributable to the initial retrieval attempts both groups carried out during the first sessions) and have included more careful wordings and reservations in the discussion.

Lines 511-516:

There are four major take-aways from this study, namely that 1) retrieval effort predicts accuracy over time, 2) retrieval effort decreases over time, 3) confidence increases over time mainly for incorrect memories when memories are repeated, and 4) retrieval effort mediates the relation between confidence and accuracy. *However, there were also methodological constraints, leading to smaller than expected effects of time and repetition, that also potentially limits generalizability.*

Lines 575-621:

The increased retrieval ease for the no-repetition group at T2 is not likely due to spontaneous repetition among these participants, as they scored low on the two questions about time spent reflecting on the event, nor due to selective reporting of easily retrieved memories (see Koriat & Goldsmith, 1996), as there was no significant reduction in the number of total unique details reported between T1 and T2 (see Fig 1). *Instead we see three plausible explanations for this effect: a) repeated retrieval opportunities as T1, b) context-dependent learning effects, and c) switch in grain-size of reported details.*

*The first explanation—repeated retrieval opportunities at T1—is probably the most important. That is, the participants were allowed to retrieve their memories of the event directly after seeing it, as they were interviewed about the event. They did this several times, first in the free recall session and again in the cued recall session. In addition, there was a third retrieval opportunity, namely during the confidence ratings. During this task, the experimenter read aloud details that the witness had said, and thus allowed the participant to elaborate upon each mentioned detail. This abundance of retrieval attempts likely helped participants to consolidate their memory of the event, leading to minor forgetting at T2 for both groups. Although this could have likely been avoided with a between-group design in which one group were only tested at T2, a within-group was the optimal choice to follow the development of effort cues over time, given the variance in use of effort cues between individuals (see Gustafsson et al., 2021, but see also T1 in Fig 4 above).*

*The second explanation—context-dependent learning (Godden & Baddeley, 1975)—suggests a more successful retrieval when the retrieval takes place at the same location as the encoding of the event. Our participants were interviewed in the same experimental room during both sessions, so it is possible that this context facilitated their retrieval, minimizing potential forgetting effects during the retention interval.*

*The third explanation is a shift in grain-size of the details. Koriat and Goldsmith (1996) suggests that people can not only decide to withhold or report a memory, but also shift the level of detail with which the memory is reported. Thus, a piece of clothing could both be described with a high level of detail (“a blue zip up jacket with green stripes and a hood”) or a low level of detail (“a jacket”). In this study we did not code for grain-size, so it is possible that the repetition group remembered things in more detail than did the no-repetition group. We encourage researchers to examine this in future studies, and as our data is openly available, suggest examinations also in our data.*

*We suggest that these explanations also likely led to a smaller overall effect of the repetition manipulation, due to floor effects.*

*In addition to expecting an easier overall retrieval at T2 for the repetition group, we also expected* an increased retrieval ease incorrect statements compared to correct statements, again due to expected floor-effects for correct memories. The findings indeed indicate a greater retrieval ease for incorrect memories (see Fig 4), but the time-accuracy interaction was not statistically significant*. If this effect is not due to changes in grain-size of reporting, one might dismiss* it as a too weak manipulation *of repetition*. However, we believe that this showcases something more important, namely that all memories are not as fragile and easily manipulated as the memory research field might sometimes give the impression of (see also Knutsson et al., 2011; Odinot et al., 2013). Finally, although it is worth pointing out again that the effort cues still predicted accuracy at T2 despite the general increase in retrieval ease (see Fig 4), a continued decrease over time will likely lead to a point wherein accuracy can no longer be distinguished by retrieval effort cues. Thus, examining effects of longer retention intervals with repetition is an avenue for future research.

We have also added a comment on grain size in the discussion, highlighting its potential role in explaining the results – see response to comment #2.

9. I wondered why “hedges” were considered an index of effort. They seem, intuitively, to be markers of uncertainty (and therefore conceptually related to confidence). Can the authors elaborate on why these are best viewed as cues associated with effort rather than cues indicating uncertainty?

**Response:** The way we see it, uncertainty and effort are intimately linked, and can be seen as two sides of the same coin. Just like a pause or a filler can be seen as a hesitation and uncertainty, so can a hedge. This relates to memory strength and the underlying idea that memories that are strongly manifested will be easier to retrieve and hence, carry greater certainty. The difference between hedges and confidence is that we see confidence as a more conscious expression of certainty, whereas a pause or a hedge is more automatic.

10. The claim that confidence judgements seem to be especially unreliable when made a considerable time after the event (line 120) needs some support (e.g., citations). Typically, the predictive value of confidence varies according to the interval between the decision/retrieval effort and confidence, rather than the interval between encoding and confidence (see Palmer et al., 2013; Sauer et al., 2010; Wixted et al., 2016, for examples). It’s true that overconfidence can increase with retention interval, but that’s not to say that confidence is unreliable. Perhaps this also depends on whether one looks at the recall or recognition literatures?

**Response:** We have rephrased this sentence and clarified citations. We have also clarified distinctions between recall and recognition evidence. See response to comment #3.

11. On line 469, the authors conclude that “retrieval effort decreased over time…”. Given that accuracy did not, this poses a conundrum: How were participants able to maintain accuracy, despite decreased effort, as retention interval increased? I think the answer probably relates to the operationalization of retrieval effort in this claim. Specifically, I’m not sure that we can equate “retrieval efforts cues” to retrieval effort. Thus, although the presence of retrieval effort cues might decrease over time, I’m not sure it can be concluded that retrieval effort decreased. Cues are just that: Cues. They may be indicative of underlying effort, but they are not direct indexes of the underlying construct. This is, in some sense, a wording issue but it is conceptually important.

**Response:** We agree that retrieval effort and retrieval effort cues can not be completely equated. We have therefore changed the wordings throughout the text to reflect this. However, as to the conundrum, we do not see a big problem. We would argue that accuracy was maintained over time as *both* correct and incorrect memories became easier to retrieve. This largely kept the relative difference in effort between retrieving correct and incorrect memories constant (as evident from Fig 4) – although there was also a tendency for a decreased difference between the two.

12. * On a related note: If accuracy doesn’t change over time, but indices of retrieval effort decreases over time, it seems there might be some need to qualify (if only slightly) the conclusion that retrieval effort predicts accuracy over time (i.e., the two must, to some extent, start to dissociate).

**Response:** Yes we agree and have added the following to explicate this.

Lines 526-528:

The results thus suggest that retrieval effort can be a reliable predictor of memory accuracy over extended retention intervals *(however, see also section “Retrieval ease decreases over time” below).*

Lines 617-621:

*Finally, although* it is worth pointing out again that the effort cues still predicted accuracy at T2 despite the general increase in retrieval ease (see Fig 4), *a continued decrease over time will likely lead to a point wherein accuracy can no longer be distinguished by retrieval effort cues.* *Thus, examining effects of longer retention intervals with repetition is an avenue for future research.*

**Reviewer 2**

13. The authors should include an important unmentioned explanation for why confidence increases over time: post-identification feedback (e.g., Wells & Bradfield, 1998)

**Response:** We have now added information about the influence of feedback on confidence in testimonies, and included a citation for post-identification feedback.

Lines 177-183:

*Although memory strength/ease of retrieval largely plays a large part in memory recall, there are also other factors that influences judgments about memories, and willingness to report them. Key factors can be grouped around effects on the belief in a memory’s accuracy. For example, leading questions (e.g. Loftus & Palmer, 1974) and post-event feedback (Gurney et al., 2014; for identification research, see meta-analysis by Douglass & Steblay, 2006) may bolster one’s belief that a memory is correct, and increase the chances that it is reported (and subsequent confidence judgment).*

14. The authors should also consider including reference to retrieval-enhanced suggestibility (e.g., Chan, Wilford, & Hughes, 2012).

**Response:** We have now added information about the potential effect of retrieval-enhanced suggestibility, lines 145-150:

One important form of repetition that strengthens memory is repeated retrieval of a memory, generally known as the testing effect. This has consistently been shown to lead to better retention and retrieval compared to a mere repeated exposure (e.g. Karpicke & Roediger, 2008; see Rowland, 2014 for a meta-analysis), (e.g. Karpicke & Roediger, 2008; see Rowland, 2014 for a meta-analysis) *although retrieval can also increase belief in false information if presented after the retrieval attempt (e.g. Chan, Wilford, & Hughes, 2012).*

15. One potential issue with these data is that both groups showed decreased retrieval effort at Time 2, even though the manipulation check items indicated that the repetition group did rehearse their memories more so than the control group. The authors attribute this null finding to context-dependent learning in which the control group’s recall was boosted by being in the same environment at Time 1 and Time 2. This hypothesis bears pursuing because the repetition group also benefitted (presumably) from context-dependent learning. Is it the authors’ contention that context-dependent learning retrieval effort produced a floor effect of retrieval effort such that repetition couldn’t have any additional effect? I wonder if these data would be more useful with the addition of another experiment where the context-dependent learning explanation can be eliminated.

**Response:** After a discussion, we have concluded that it is likely that the retrieval and confidence rating during the T1 interview in large part account for the similarity in retrieval effort at Time 2, together with context-dependent learning - and potentially also by differences in grain-size reporting. Indeed, we do believe that this likely contribute to the small additive effect of our repetition manipulation. Please see our response to comment #8 for a more extensive explanation, including changes to the manuscript.

16. I was also surprised that there was no effect of time on number of accurate statements or total number of accurate details, nor was there any interaction between repetition and time (page 14). This seems counterintuitive to previous research showing that memory decays over time. Using this finding to argue that “confidence can remain a stable predictor over time” (page 24) is unwise because this seems to be a particularly unique scenario in which there was no memory loss over two weeks.

**Response:** As argued above, we believe the lack of forgetting was largely due to the retrieval attempts given at T1 for both groups. Although this does not demonstrate the classic forgetting effect, we don’t see it as unlikely that a witness will reflect back on an event shortly after it occurs, and thus the design still carries some relevance for real-life scenarios. That said, we agree with your assessment that this is not a general finding in psychology, and have changed the sentence to be in accordance with the reasoning:

Lines 631-633:

This is an interesting result, because it *would* suggest that we should effectively trust people’s confidence for correct memories, as these judgments were relatively stable over time and across repetition *in our study*.

Lines 644-646:

*Some reservations to these suggestions are necessary however, as we did not observe the typical forgetting effects over time, which could indicate that these results are not representative of general situations (see also “Limitations”).*

17. One other issue is that the authors dropped non-word fillers from predictive analyses examining the effect of time and repetition on the relationship between accuracy and retrieval effort. Given that this DV was significant (albeit in the opposite direction than predicted), it seems like some additional analyses are warranted. Could it not have been included in the model as a reciprocal predictor, as in, fewer non-word fillers predicting accuracy? Couldn’t this significant predictor explain some of the “leftover variance” (page 24)?

**Response:** In the Supporting information, we present individual mediation analyses for each effort cue (see S2 Table). Non-word fillers mediated less than 0.01 % of the relation between Accuracy and Confidence, so we deem it unlikely that it explains the remaining variance.

18. I think the authors need to be more cautious in their conclusions about believing confident witnesses (page 23). Their argument is only appropriate if intervening influences are not present (e.g., post-identification feedback, influence from co-witnesses).

**Response:** We agree and have now qualified our conclusions in the Limitations section about generalizing our findings to real eyewitnesses. See also our response to comment #16.

Lines 726-727:

*Moreover, our participants were not explicitly exposed to influences from co-witnesses, post-event information or direct forms of misinformation.*

19. I would like the authors to address how to use these data in the field. Namely, how could information about hedges and delays help individual jurors or investigators evaluate particular witnesses? Is there some criterion of hedge/delay behavior beyond which witnesses become untrustworthy? Probably not, but it would be useful to have the authors speculate on what an individual evaluator should do with this information.

**Response:** We have added such a speculation on lines 555-566:

*Given the consistent effort-accuracy relation, a reasonable question to ask is how to use this knowledge in the field as a practitioner. Because there appears to be some variation between individuals in expressing effort (see e.g. “T1” in Fig 4) we suggest that this knowledge is as of now best used carefully, ideally in conjunction with other corroborating evidence, such as physical evidence or other eyewitness reports. A starting point could be to judge statements without hedges or delays as correct, which is supported by the unstandardized odds ratios in Table 2,* *which show that each hedge should decrease the chance of accuracy by 29%, and each delay should decrease the accuracy by about 16 %. Such a method has actually shown some success in improving judgment accuracy of eyewitness testimonies (see Gustafsson et al., 2021), and is similar to the recommendation given by Wixted and Wells (2017) for identification research, where they suggest that highly confident witnesses should generally be believed (given “pristine” conditions).*

20. Page 12 – there are kappa values for the second and third exact overlap percentages, but not the first (line 268)

**Response:** Correct. This is actually intentional on our part, because we find no sensible way to calculate kappa values for coding of statements. Kappa value calculations require both “yes” and “no” responses, in addition to agreement/disagreement between coders. Whereas we are able to calculate agree/disagree on “yes” responses (i.e. at least one coder codes an utterance as a statement), we cannot calculate agree/disagree on “no” responses. Instinctively, all utterances that is not coded by the coders as statements could indirectly be seen as *not statements*, but because these utterances are not separated into specific “non-statements”, we cannot calculate how many of them there are. For example, a twenty-word passage could theoretically be divided into two, three, seven or perhaps even eighteen smaller segments. Because of this, we only present the exact overlap, which is also a stricter measure than Cohen’s kappa.
